# Effects of physical activity on the progression of diabetic nephropathy: a meta-analysis

**DOI:** 10.1042/BSR20203624

**Published:** 2021-01-05

**Authors:** Zixin Cai, Yan Yang, Jingjing Zhang

**Affiliations:** National Clinical Research Center for Metabolic Diseases, Metabolic Syndrome Research Center, Key Laboratory of Diabetes Immunology, Ministry of Education and Department of Metabolism and Endocrinology, The Second Xiangya Hospital of Central South University, Changsha 410011, Hunan, China

**Keywords:** Diabetic nephropathy, Lifestyle, Physical activity

## Abstract

Background: Diabetic nephropathy (DN) is an important microvascular complication of diabetes. Physical activity (PA) is part of a healthy lifestyle for diabetic patients; however, the role of PA in DN has not been clarified. Our aim was to conduct a meta-analysis to explore the association between PA and DN risk.

Methods: PubMed, Embase, Cochrane Library and Web of Science were systematically searched for articles examining PA in diabetic patients and its effect on renal function. Standardized mean differences (SMDs) and odds ratios (ORs) with 95% confidence intervals (CIs) were calculated. The study protocol is registered with PROSPERO (CRD42020191379).

Results: A total of 38991 participants were identified from 18 studies. The results indicated that PA was associated with increases in the glomerular filtration rate (SMD = 0.01, 95% CI = [0.02–0.17]) and decreases in the urinary albumin creatinine ratio (SMD = −0.53, 95% CI: −0.72 to −0.34), rate of microalbuminuria (OR = 0.61, 95% CI = [0.46–0.81]), rate of acute kidney injury (OR = 0.02, 95% CI = [0.01–0.04]), rate of renal failure (OR = 0.71, 95% CI = [0.52–0.97]) and risk of DN in patients with Type 1 diabetes (OR = 0.67, 95% CI = [0.51–0.89]).

Conclusions: This meta-analysis indicated that PA is effective for improving DN and slowing its progression; however, more high-quality randomized controlled trials are required on this topic.

## Introduction

Diabetes mellitus (DM) is a highly prevalent metabolic disease that has caused considerable humanistic and financial burdens to society and families [1]. According to estimates from the International Diabetes Federation (IDF) diabetes atlas, 463 million adults are currently living with diabetes worldwide, indicating that 1 in 11 adults (20–79 years old) have diabetes [2]. Moreover, 35% of people with diabetes may develop end-stage renal disease after 5 years and 18% die from diabetic nephropathy (DN) after 20 years of disease duration [3]. The high incidence of diabetes has placed a heavy burden on individuals, families and society. In general, strict glycaemic control could result in better outcomes in DN patients. An observational study showed that both higher (> 8.0%) and lower (< 6.5%) levels of HbA1c were negatively correlated with DN [4]. These findings suggest that optimal control of glycaemic levels is an essential measure for DN. The complicated pharmacokinetics of antidiabetic drugs leads to difficulties in glycaemic control in chronic kidney disease patients [5]. Indeed, the existence of impaired renal function is an important risk factor for severe hypoglycaemia [6], which is partly due to impaired gluconeogenesis in the kidney [7]. Additionally, both decreased glucose filtration and increased inflammation-induced insulin resistance result in hyperglycaemia in patients with kidney disease [8]. DN is a disease that increases the predisposition to hyperglycaemic or hypoglycaemic circumstances and other side effects; therefore, drug treatments for these patients should be chosen very cautiously. Above all, it is urgent to explore low-cost and safe treatment steps to decrease the incidence and mortality rate of DN.

Physical activity (PA) has been universally acknowledged as a healthy lifestyle intervention to prevent complications in diabetes and is recommended in the ‘Standards of Medical Care in Diabetes’ for DM patients by the American Diabetes Association (ADA) [9]. PA is recommended for all adults, including those with chronic conditions, to achieve health benefits [10]. However, there are conflicting results regarding the role of PA in DN. Some studies demonstrated that there was no correlation between PA and the development or progression of DN [11], while other studies demonstrated a significantly lower risk of DN in diabetic patients who actively participated in PA [12,13]. Because of the inconsistent conclusions among these previous studies and the relatively small number of cases in each study, the role of PA in DN is still uncertain. Therefore, a meta-analysis was performed in the present study to verify whether DN patients could improve their renal function by participating in PA.

We conducted a meta-analysis to confirm the efficacy of PA in terms of DN progression, and the results may be helpful in DN management. We hope to determine whether PA is beneficial to the renal function of DN patients and provide PA guidelines for patients with DN.

## Methods

### Registration of the review protocol

This meta-analysis was conducted according to PRISMA (Preferred Reporting Items for Systematic Reviews and Meta-Analyses) guidelines [14]. This systematic review was prospectively registered in PROSPERO (CRD42020191379) and is available at http://www.crd.york.ac.uk/PROSPERO/display_record.php?ID=CRD42020191379.

### Literature search strategy

Electronic databases, including PubMed, Embase, Cochrane Library and Web of Science, were thoroughly searched from inception to July 2020. The following keywords were used as search terms in variable combinations: (Exercises OR Physical Activity OR Activities, Physical OR Activity, Physical OR Physical Activities OR Exercise, Physical OR Exercises, Physical OR Physical Exercise OR Physical Exercises OR Acute Exercise OR Acute Exercises OR Exercise, Acute OR Exercises, Acute OR Exercise, Aerobic OR Aerobic Exercise OR Aerobic Exercises OR Exercises, Aerobic OR Exercise Training OR Exercise Trainings OR Training, Exercise OR Trainings, Exercise), AND (Nephropathies, Diabetic OR Nephropathy, Diabetic OR Diabetic Nephropathy OR Diabetic Kidney Disease OR Diabetic Kidney Diseases OR Kidney Disease). We also manually searched for additional papers on PA and DN in the reference lists of the reviewed articles.

### Selection criteria

After duplicates were removed, all the articles for inclusion in this meta-analysis met the following criteria: (1) patients had Type 1 or 2 DN or diagnosed DM; (2) the interventions in the studies included PA and controls; (3) at least one of the following outcome measures was reported: serum creatinine, glomerular filtration rate (GFR), urinary albumin creatinine ratio (UACR), microalbuminuria, acute kidney injury (AKI) and renal failure; and the (4) article was published in the English language. Studies that met the following criteria were excluded: (1) case reports, case series, letters to editors and review articles; (2) study subjects other than humans; (3) incomplete or incorrect data; and (4) irrelevant or no outcomes.

### Data extraction

Two authors (Z.C. and Y.Y.) independently extracted the following parameters: first author (publication year), country, age range/year, participants, DM type, outcome measures and quality score. Any disagreements were resolved by consensus or by referring to a third author (J.Z.).

### Quality assessment

Since there was no assessment method suitable for various study types (i.e., RCT, cross-sectional study and cohort study), risk of bias was assessed using the Cochrane Collaboration tool and Newcastle–Ottawa scale (NOS). To assess the validity of the included RCTs, we evaluated the risk of bias using the Cochrane Collaboration assessment criteria, while the quality of non-randomized studies was assessed with the NOS. Two independent reviewers (Z.C. and Y.Y.) performed the quality assessment, and disagreements on scores were resolved through discussion.

### Publication bias and sensitivity analysis

To test the robustness of the pooled results, we evaluated the potential of publication bias by constructing a funnel plot. We performed a sensitivity analysis by omitting each study one by one.

### Statistical analysis

Review Manager Software (RevMan V5.1, Cochrane Collaboration, U.K.) and STATA software (version 12.0, STATA Corp, College Station, TX, U.S.A.) were used for data analysis. Continuous variables were expressed as the standard mean difference (SMD) with 95% confidence interval (CI). Categorical data were expressed as the odds ratios (ORs). The SMD with its 95% CI was used to analyse the GFR, UACR and serum creatinine. Statistical analyses for dichotomized outcomes (rate of microalbuminuria, rate of DN, rate of AKI and rate of renal failure) were performed using the OR and 95% CI. The heterogeneity of the included studies was verified with the *I*^2^ statistic and Chi-squared test *P*-value. When *I*^2^>50% or *P*<0.05, a random-effects model was used; otherwise, a fixed-effects model was adopted.

## Results

### Study characteristics

**Figure 1:**
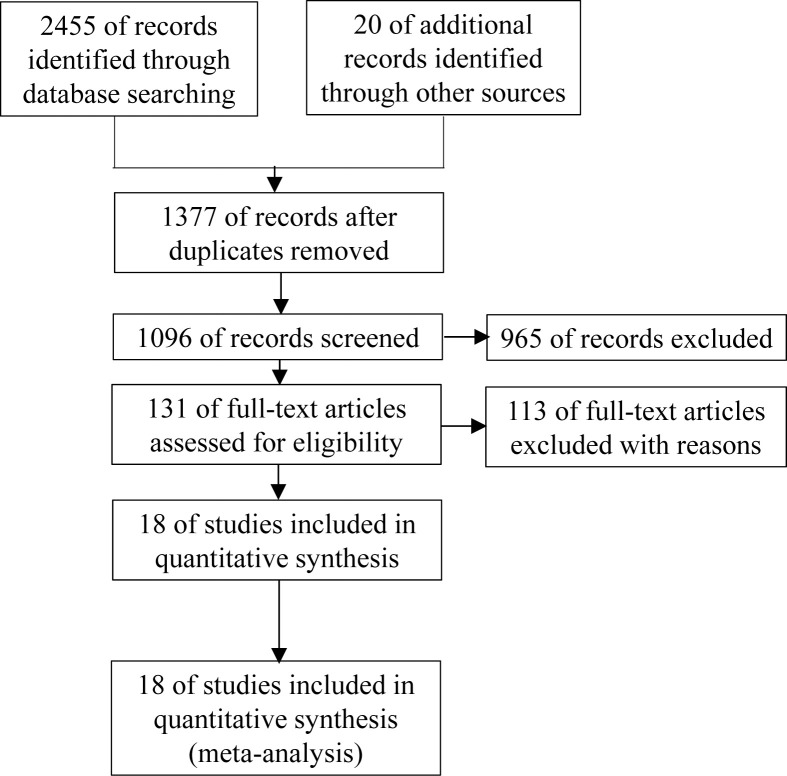
Study selection

Figure 1 demonstrates the details of the included studies in this meta-analysis. Through a search using the index words, a total of 2475 publications were initially identified. After screening the title, abstract and duplicate publications, 2344 publications were eliminated. During full-text screening, 113 studies were excluded for failing to provide qualified endpoints or complete data for our meta-analysis. Therefore, a total of 18 studies [11–13,15–29] that included 38991 patients were ultimately included in the current meta-analysis to evaluate the role of PA in DN patients.

### Characteristics of eligible studies

The major characteristics of the studies are shown in Table 1. The basic information consisted of first author (publication year), country, age range/year, participants, DM type, outcome measures and quality score. A total of 38,991 individuals from the United States, Uganda, Ethiopia, Finland, Japan, Qatar, Spain, Nepal, Serbia, Germany and Australia. In all, 14 non-RCTs and 4 RCTs were included. Eight studies analysed Type 2 diabetes, six studies analysed Type 1 diabetes, and two studies analysed both Types 1 and 2 diabetes. In addition, two studies did not specify the type of diabetes.

**Table 1:** Characteristics of eligible studies

Number	First author (publication year)	Country	Age range/year	Participants	DM Type	Outcome measures	Quality score
1	Martin Muddu (2019)	Uganda	46 ± 15	175	T1DM,T2DM	Microalbuminuria	6
2	Lemma Demissie Regassa (2019)	Ethiopia	48.31 ± 14.85	502	T2DM	AKI	8
3	Sanjay Kumar Thakur (2019)	Nepal	41–80	227	T2DM	Microalbuminuria	7
4	Hirohito Kuwata (2017)	Japan	65.5 ± 11.5	1814	T2DM	Serum creatinine;GFR;UACR	8
5	Johan Wadén (2015)	FinnDiane	37.0 ± 12.4	1390	T1DM	Diabetic nephropathy	6
6	Yiqun Chen (2015)	Durham	65+	1142	NA	Renal failur	6
7	Barbara Bohn (2015)	Germany and Austria	18–80	18028	T1DM	Microalbuminuria	6
8	Abdulbari Bener (2014)	Qatar	45.3 ± 15.0	1633	T1DM,T2DM	Diabetic nephropathy	8
9	Florentino Carral (2013)	Spain	18–60	130	T1DM	Diabetic nephropathy	7
10	JOHAN WADEN (2008)	Finnish	38.5 ± 12.3	1945	T1DM	Microalbuminuria	8
11	Gordana Lazarevic (2007)	Serbia	54.8 ± 7.3	30	T2DM	UACR	6
12	Kempei Matsuoka (1991)	Japan	NA	24	NA	Serum creatinine	6
13	ANDREA M. KRISKA (1991)	U.S.A.	8–48	628	T1DM	Diabetic nephropathy	8
14	I Ala-Houhala (1990)	Finland	32 ± 9	12	T1DM	GFR;UACR	5
15	J. I. Blomster (2013)	20 countries	65.8 ± 6.4	11140	T2DM	Creatinine clearance	RCT
16	David J. Leehey (2016)	U.S.A.	49–81	32	T2DM	UACR;GFR	RCT
17	E. S. Nylen (2015)	U.S.A.	62 ± 2.1	128	T2DM	Serum creatinine;GFR	RCT
18	David J Leehey (2009)	U.S.A.	NA	11	T2DM	Serum creatinine;GFR;UACR	RCT

### Quality assessment of included studies

**Figure 2:**
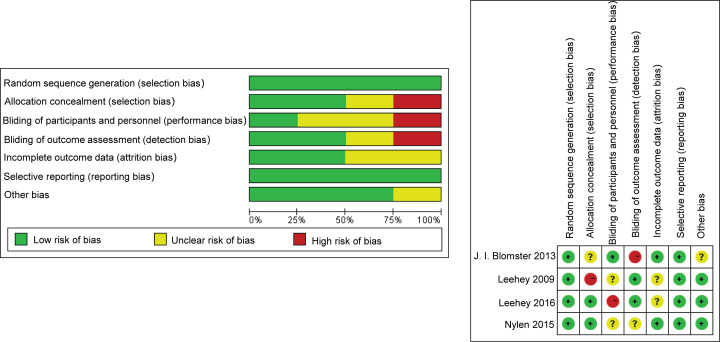
Risk of bias graph of the included trials Review authors’ judgments about the risk of each bias item for each included study (**A**) and review authors' judgments about the risk of each bias item presented as percentages across all included studies (**B**).

Figure 2 shows the risk bias of the included RCTs. The included studies showed great variations in overall quality. The studies included in this review varied in their study design, methodology and structure. Of the 4 studies identified for analysis in the present study, 1 was classified as high quality and 3 were classified as low quality. The risk of bias of the non-RCT trials is displayed in Table 1.

### Outcomes

#### Effects of PA on GFR

**Figure 3:**
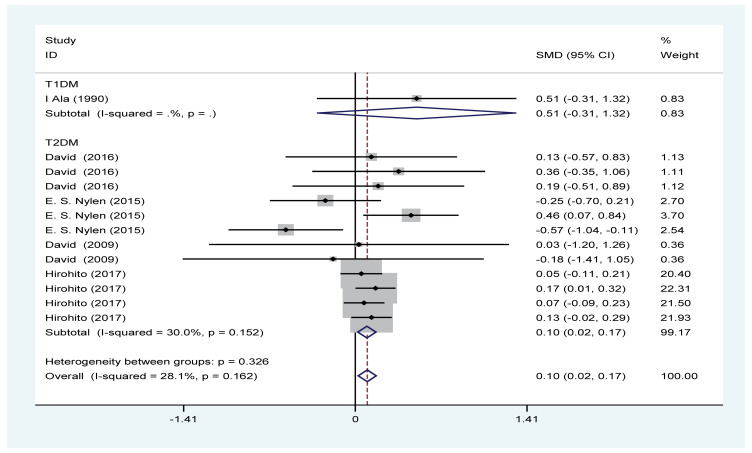
Forest plot evaluating effects of exercise on change in eGFR

Five trials with a total of 2137 patients (PA group = 1730; control group = 407) showed the effect of PA on GFR. Based on the *I*^2^ test value (*I*^2^ = 28.1%) and Chi-squared test *P*-value (*P*=0.162), the fixed-effects model was applied to analyse GFR. The pooled results showed that the GFR was increased in the PA group versus the control group (SMD = 0.1, 95% CI: 0.02–0.17) (Figure 3).

#### Effects of PA on UACR

**Figure 4:**
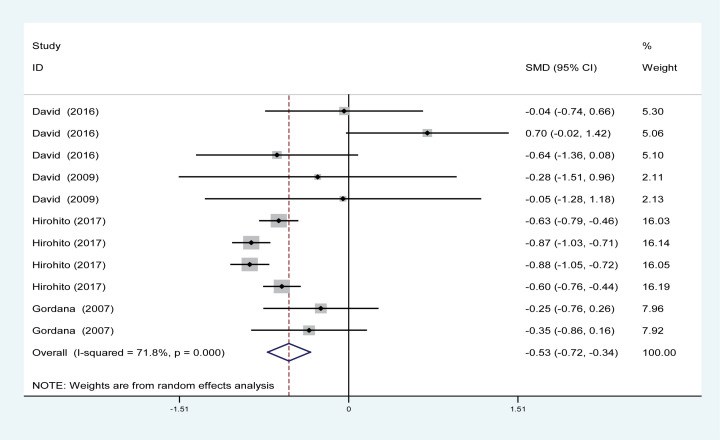
Forest plot evaluating the effects of exercise on change in UACR

Four trials involving 1917 patients (PA group = 1620; control group = 297) showed the effect of PA on UACR. Based on the *I*^2^ test value (*I*^2^ = 71.8%) and Chi-squared test *P*-value (*P*<0.001), the random-effects model was utilized to analyse UACR. The pooled results showed that the UACR was decreased in the PA group versus the control group (SMD = –0.53, 95% CI: –0.72 to –0.34) (Figure 4).

#### Effects of PA on the rate of DN

**Figure 5:**
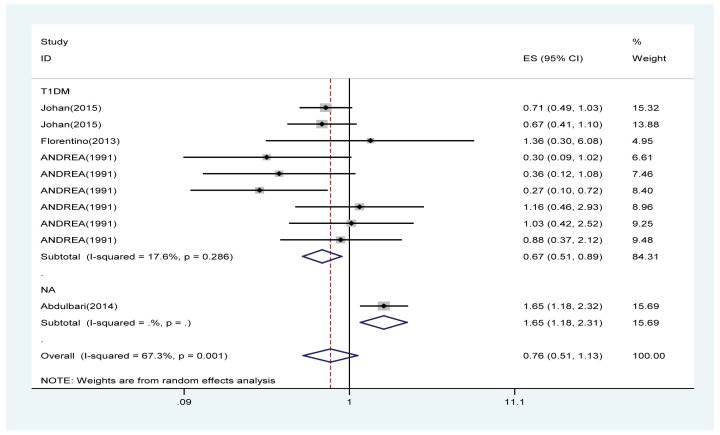
Forest plot evaluating the effects of exercise on the rate of DN

Four trials with a total of 3326 patients (PA group = 2473; control group = 853) showed the effect of PA on the rate of DN. According to the *I*^2^ test value (*I*^2^ = 67.3%) and Chi-squared test *P*-value (*P*=0.001), we analysed the rate of DN using the random-effects model. The overall pooled results showed that the rate of DN was no associated with PA. The subgroup analysis revealed that the risk of DN in T1DM obviously decreased in the PA group compared with the control group (OR: 0.67, 95% CI: [0.51–0.89]) while the risk of DN increased for certain types of diabetes (OR = 1.65, 95% CI: [1.18–2.31]; Figure 5).

#### Effect of PA on serum creatinine

**Figure 6:**
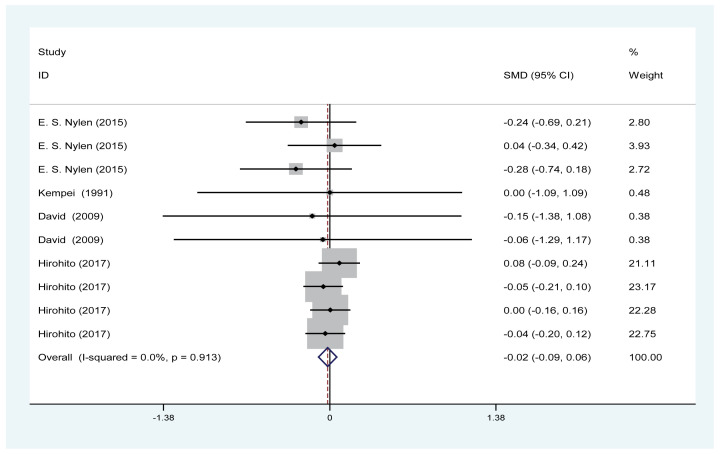
Forest plot evaluating the effects of exercise on changes in serum creatinine

Four trials involving 2094 patients (PA group = 1710; control = 384) reported the effect of PA on serum creatinine. According to the *I*^2^ test value (*I*^2^ = 0%) and Chi-squared test *P*-value (*P*=0.913), the fixed-effects model was applied to analyse serum creatinine. No significant difference in serum creatinine was found in the pooled results between the PA and control groups (SMD = −0.02, 95% CI: [−0.09 to 0.06]; Figure 6).

#### Effects of PA on the rate of microalbuminuria

**Figure 7:**
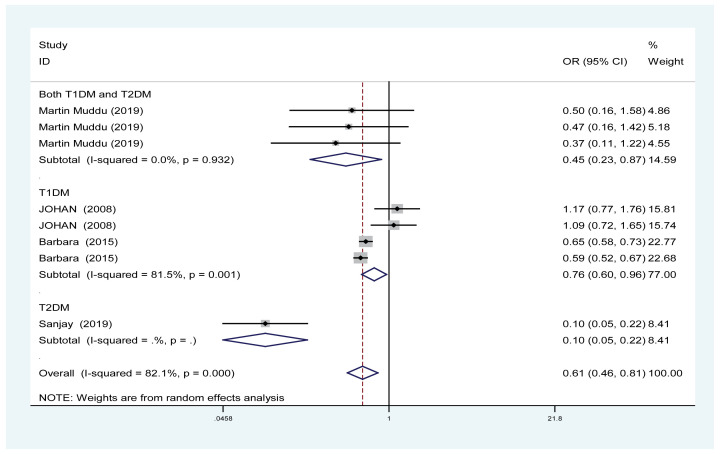
Forest plot evaluating the effects of exercise on the rate of microalbuminuria

Four trials involving 15288 patients (PA group = 5867; control group = 9421) showed the effect of PA on the rate of microalbuminuria. Based on the *I*^2^ test value (*I*^2^ = 82.1%) and Chi-squared test *P*-value (*P*<0.001), we analysed the rate of microalbuminuria using the random-effects model. The rate of microalbuminuria was significantly decreased in the PA group compared with the control group (OR: 0.61, 95% CI: [0.46–0.81]; Figure 7).

#### Effects of PA on the rate of AKI

**Figure 8:**
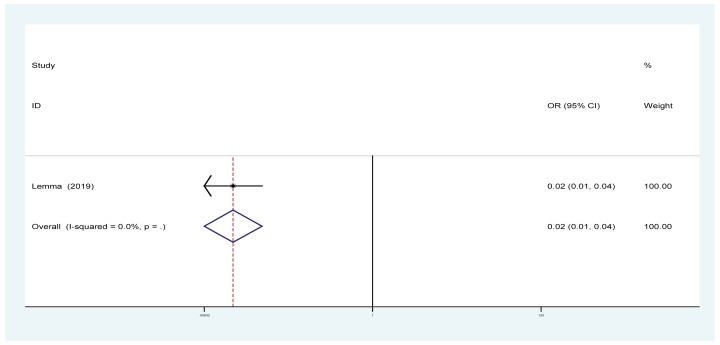
Forest plot evaluating the effects of exercise on the rate of AKI

One trial with a total of 502 patients (PA group = 371; control group = 131) showed the effect of PA on the rate of AKI. The pooled results showed that the rate of AKI was obviously decreased in the PA group compared with the control group (OR: 0.02, 95% CI: [0.01–0.04]; Figure 8).

#### Effects of PA on the rate of renal failure

**Figure 9:**
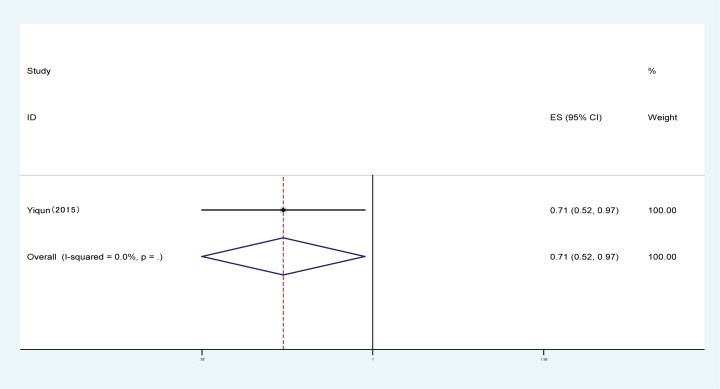
Forest plot evaluating the effects of exercise on the rate of renal failure

One trial with a total of 1142 patients showed the effect of PA on the rate of renal failure. The pooled results showed that the rate of renal failure was obviously decreased in the PA group compared with the control group (OR: 0.71, 95% CI: [0.52–0.97]; Figure 9).

### Publication bias and sensitivity assessment

**Figure 10:**
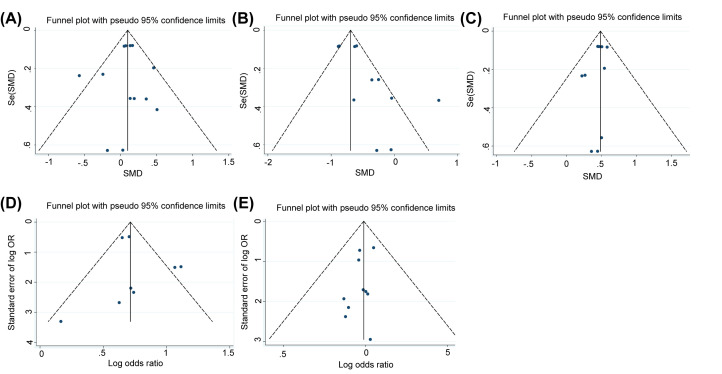
Publication bias analysis based on a funnel plot of eGFR (**A**) UACR (**B**) serum creatinine (**C**) the rate of microalbuminuria (**D**) and the rate of DN (**E**)

**Figure 11:**
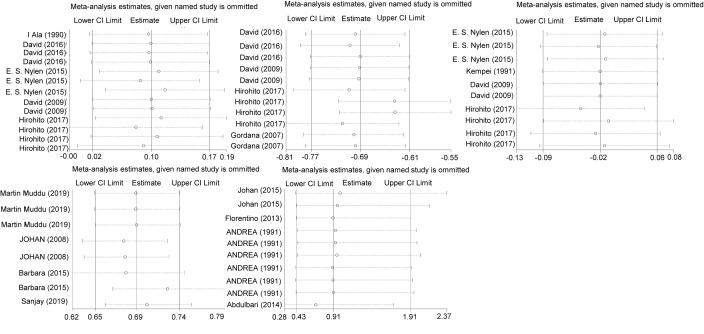
Sensitivity analysis for the effects of PA on eGFR (**A**), UACR (**B**), serum creatinine (**C**), the rate of microalbuminuria (**D**) and the rate of DN (**E**)

The funnel plots for GFR, serum creatinine, rate of microalbuminuria and rate of DN showed a symmetric distribution, suggesting a lack of publication bias. However, the funnel plot for UACR revealed an asymmetric distribution, suggesting publication bias (Figure 10). In the sensitivity analysis, the pooled result was stable (Figure 11).

## Discussion

### Association between PA and DN

PA effectively improved the estimated GFR and decreased the UACR in DN patients (Figures 3 and 4); however, our results revealed that PA did not show obvious efficacy in reducing serum creatinine, which may be due to the diverse types of research studies. Compared with serum creatinine alone, GFR is the gold standard for accurately measuring global renal function [30]. Interestingly, PA reduced the rate of microalbuminuria, the risk of DN in patients with T1DM, the rate of AKI and the rate of renal failure in DM patients (Figures 5 and 7–9). This meta-analysis indicates the protective effective role of PA in DN.

### Mechanisms underlying the effect of PA on DN

Several plausible biological mechanisms have been proposed to explain the possible relationship between PA and DN. In both T1DM and T2DM, hyperglycaemia is a major determinant of the progression of DN [31]. A negative relation between HbA1c levels and PA was found in a British study [32]. These results indicated that PA manifested its effects through glycaemic control.

Blood pressure reduction via antihypertension drug treatment leads to beneficial effects on renal outcomes [33]. Recent systematic reviews of intervention studies showed that PA-focused lifestyle interventions can be effective in the prevention of hypertension [34].

An epidemiological study showing that patients with DN often have multiple lipoprotein abnormalities [35] indicated that disruptions in lipid metabolism may promote glomerular and tubulointerstitial injury. Lipids are now believed to have a direct role in the pathogenesis of chronic kidney disease and therefore probably contribute to the high risk of cardiovascular morbidity and mortality associated with DN [36]. Strong experimental evidence has shown that PA could decrease the risk of developing dyslipidaemia through weight loss, and the current study showed that PA may have a beneficial effect on the lipid profile [37].

Endothelial function was more severely impaired in patients with overt nephropathy than in those with incipient nephropathy [38]. Investigators confirmed the positive association between endothelial function and PA [39]. Prolonged sedentary activity has been associated with greater insulin resistance, lack of insulin sensitivity, impaired microvascular function and increased overall morbidity and mortality [40]. Given this evidence, the detrimental effects of prolonged sedentary lifestyle may accelerate the progression of microvascular complications in persons with diabetes, including the development of DN.

PA exerts numerous beneficial effects on glycaemic control, lipid profiles, blood pressure and endothelial function, and the evidence above indicates that the positive benefits of a physically active lifestyle in the treatment of DN are substantial.

### Theoretical and practical implications

Our meta-analysis not only provides implications for practice but also offers implications for research. On the practical side, PA performed in daily life is beneficial for health and recommended by the World Health Organization. Moreover, since our review pooled 18 studies from various geographical locations and analysed a variety of PA and exercise programmes, increased PA may be applicable to people all over the world. On the research side, previous studies have not used dose–response models to show the magnitude of decreased DN risk that corresponds to an increase in the amount of total PA. Ascertaining the dose-response relationships between the intensity of PA and DN outcomes will require long-term experimentation and additional research to provide clearer guidelines.

### Limitations of our study

There were some limitations in this meta-analysis. First, since 14 of the 18 included studies were non-RCTs, we could not determine their causal relationship despite finding a correlation between PA and DN. Second, whether PA was performed and the intensity of PA were obtained by self-reports; however, the self-report format cannot precisely reflect real phenomena, especially when exercise is divided into different intensities. Third, many factors lead to heterogeneity, and such factors in the meta-analysis included the age range of the participants, the country of the participants, and other items. Therefore, additional studies are still warranted in the future to further confirm our results.

### Factors that lead to inconsistent effects of PA on DN

The possible reasons for the inconsistent relationship between PA and DN are as follows. First, definitions of the form and intensity of PA are inconsistent; for example, some studies describe activity over 8 h for 5 days per week as work while others described the amount of leisure-time physical activity (LTPA) as work. Moreover, certain studies have inquired about the number of flights of stairs climbed daily, the number of blocks walked daily, and sports participation during the past week. Second, the definition of microalbuminuria is inconsistent. Some studies defined microalbuminuria as an albumin to creatinine ratio (ACR) between 30 and 299 mg/g while other defined it as at least two of three urine samples with an albumin excretion rate between 20 and 200 pg/min. Lastly, most of the PA data are obtained from questionnaires, which increases the difficulty of developing a unifying standard.

Therefore, our meta-analysis summarized 18 articles on PA and DN and determined the relationship between PA and DN from the overall level. Moreover, suggestions are proposed for future research, such as the unification of diagnostic criteria and the precise description of exercise modes and intensities.

## Conclusions

The results of this meta-analysis indicate that PA is related to improving renal function in DN patients by increasing the GFR and reducing UACR. Moreover, PA decreased the rate of microalbuminuria, the rate of DN in patients with T1DM, the rate of AKI and the rate of renal failure. More studies performing detailed quantifications of total PA will help identify a more precise estimate for different levels of PA.

## Highlights

- PA was associated with an increased glomerular filtration rate (GFR) in diabetes patients.
- PA decreased the urinary albumin creatinine ratio (UACR) in diabetes patients.
- PA decreased the rate of microalbuminuria in diabetes patients.
- PA decreased the rate of diabetic nephropathy in Type 1 diabetes patients.

## Data Availability

All data generated or analysed during the present study are included in this published article.

## Abbreviations

CI: confidence interval
DN: diabetic nephropathy
NOS: Newcastle–Ottawa scale
OR: odds ratio
PA: physical activity
SMD: standardized mean difference
